# Mammalian MicroRNA Prediction through a Support Vector Machine Model of Sequence and Structure

**DOI:** 10.1371/journal.pone.0000946

**Published:** 2007-09-26

**Authors:** Ying Sheng, Pär G. Engström, Boris Lenhard

**Affiliations:** 1 Computational Biology Unit, Bergen Center for Computational Science, University of Bergen, Bergen, Norway; 2 Sars International Centre for Marine Molecular Biology, University of Bergen, Bergen, Norway; 3 Programme for Genomics and Bioinformatics, Department of Cell and Molecular Biology, Karolinska Institutet, Stockholm, Sweden; National Cancer Institute at Frederick, United States of America

## Abstract

**Background:**

MicroRNAs (miRNAs) are endogenous small noncoding RNA gene products, on average 22 nt long, found in a wide variety of organisms. They play important regulatory roles by targeting mRNAs for degradation or translational repression. There are 377 known mouse miRNAs and 475 known human miRNAs in the May 2007 release of the miRBase database, the majority of which are conserved between the two species. A number of recent reports imply that it is likely that many mammalian miRNAs remain to be discovered. The possibility that there are more of them expressed at lower levels or in more specialized expression contexts calls for the exploitation of genome sequence information to accelerate their discovery.

**Methodology/Principal Findings:**

In this article, we describe a computational method-mirCoS-that uses three support vector machine models sequentially to discover new miRNA candidates in mammalian genomes based on sequence, secondary structure, and conservation. mirCoS can efficiently detect the majority of known miRNAs and predicts an extensive set of hairpin structures based on human-mouse comparisons. In total, 3476 mouse candidates and 3441 human candidates were found. These hairpins are more similar to known miRNAs than to negative controls in several aspects not considered by the prediction algorithm. A significant fraction of predictions is supported by existing expression evidence.

**Conclusions/Significance:**

Using a novel approach, mirCoS performs comparably to or better than existing miRNA prediction methods, and contributes a significant number of new candidate miRNAs for experimental verification.

## Introduction

MicroRNAs (miRNAs) are an abundant class of ∼22 nt long endogenous non-protein-coding RNAs that function by binding to target sites on 3′-UTRs of messenger RNAs (mRNAs) to repress translation or mediate mRNA degradation (reviewed in [1]). Mature miRNAs are synthesized from longer 70–100 nt precursors (pre-miRNAs), each of which forms a hairpin structure that contains one or two mature miRNAs in either or both of its arms. Thus far, a total of 4584 miRNAs have been identified and reported to miRBase (release 9.2, May 2007), a database that stores experimentally validated miRNAs and their homologs [2], [3]. Several recent observations indicate that miRNAs, in general, may be essential for organisms to differentiate into multiple cell- and tissue types and/or to keep cells in a particular differentiation state [4]. miRNA target prediction in mammals indicates that ∼10–30% of protein-coding genes may be under control of currently known miRNAs [5], [6]. This number may still increase because many additional miRNAs have been predicted. There are currently 475 human miRNAs and 377 mouse miRNAs in miRBase, but recent studies have suggested that the number of miRNAs in a vertebrate genome can be as many as 800–1000 [7], [8]. The high number of miRNA genes, their diverse expression patterns [9]–[12] and the abundance of potential miRNA targets suggest that miRNAs are likely to be involved in a broad spectrum of human diseases. Indeed, components required for miRNA procession and/or function have been implicated in fragile X mental retardation [13], DiGeorge syndrome [14], and cancer [15]. Lu et al. [16] demonstrated recently that miRNAs can indeed be developed into potent cancer markers.

As miRNAs are likely to play a central role in development and also in disease, it is important to understand their function. An important step towards this would be to assemble a complete catalogue of miRNA genes. Experimental cloning efforts have successfully identified highly expressed miRNAs from various tissues [9], [11], [17]–[32]. However, cloning methods are highly biased towards miRNAs that are abundantly and/or ubiquitously expressed. On the other hand, computational prediction of miRNAs could become a powerful aid for finding tissue-specific or lowly expressed miRNAs. A number of computational methods for miRNA prediction have been described and appear to complement each other because they take different approaches to miRNA prediction (reviewed in [33]).

Support vector machines (SVMs) are machine learning algorithms widely used to solve classification problems. A SVM assigns an object to one of several classes based on a set of input features associated with the object. In bioinformatics, superiority of SVMs over other classification methods has been shown for prediction of DNA-binding proteins [34], gene function [35] and protein subcellular localization [36].

Here we describe a method-mirCoS-to predict conserved miRNAs in mammalian genomes. Being fundamental functional RNA elements, many pre-miRNAs have maintained sequence and secondary structure conservation across large evolutionary distances. Conservation can be described by several features which can serve as inputs for a SVM model. Currently, five other SVM-based miRNA prediction algorithms are available. Four of them are aimed at non-conserved miRNA prediction [37]–[40], and therefore they can not achieve sufficient specificity when applied to entire large genomes. The fifth is aimed at annotating the results from non-coding RNA prediction [41], and has better specificity, predicting about 5000 miRNAs in the human genome. However, this number is still about five times higher than recent estimates [7], [8] and may include many false positives. To improve on this, we have added a number of previously unused features, and built a composite SVM model consisting of three sequentially applied SVMs and applied it to the human and mouse genomes. We predict about 3400 human-mouse conserved candidate miRNA genes, many of which show evidence of sequence and secondary structure conservation across all vertebrates (including fish) and are supported by independent evidence not used by our prediction method. Finally, we show that mirCoS performs better than or comparably to other recent methods. Because the great majority of our predictions are novel, the method described here constitutes a worthy addition to the arsenal of computational methods aimed at completing the mammalian miRNA collection.

## Results and Discussion

### Design and validation of mirCoS-a SVM-based method for miRNA prediction

**Figure 1:**
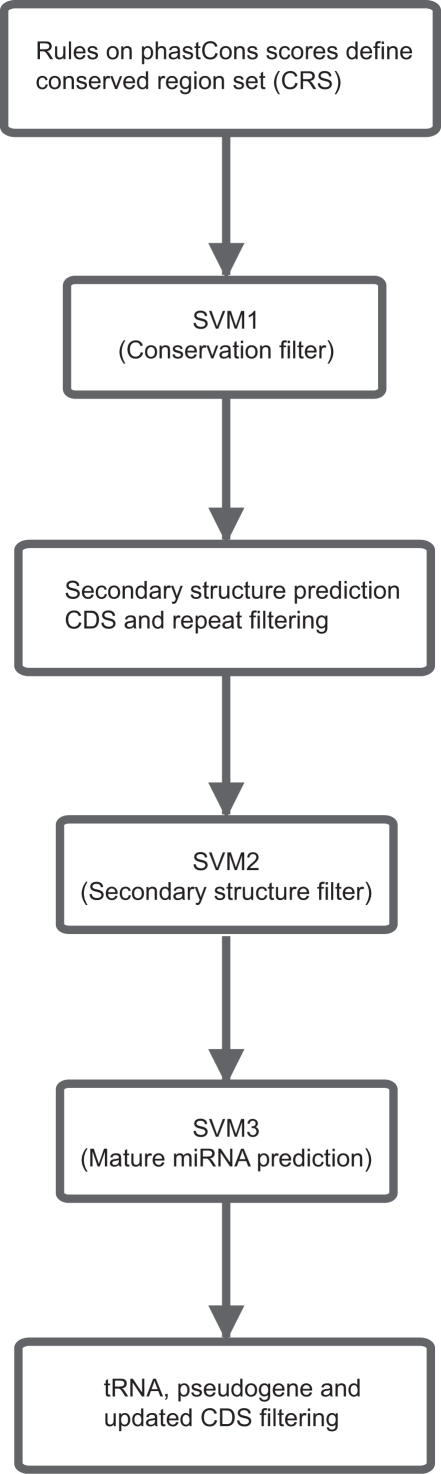
Outline of the mirCoS method.

SVMs classify objects based on a set of features for each object. With the goal of predicting mature miRNAs, we selected features describing three aspects of precursor and mature miRNAs: (1) sequence conservation of pre-miRNA, (2) secondary structure of pre-miRNA and its conservation, and (3) placement and secondary structure of mature miRNA within its pre-miRNA (Table 1). We chose to train one SVM (SVM1) for the first of these feature sets, another (SVM2) for the second, and two SVMs (one for human and one for mouse data, collectively termed SVM3) for the third feature set. We applied the SVMs sequentially, so that only candidates that were classified as positive by SVM1 were passed on to SVM2, and similarly for SVM2 and SVM3 (Figure 1). The main reason for dividing the prediction task over three SVMs was to reduce running time: the second and third feature sets require secondary structure predictions that are expensive to compute for whole genomes, and the number of objects to classify increases almost 26-fold for the third feature set, because there are many putative miRNA positions within each pre-miRNA hairpin. Additionally, the sieve effect of sequential application of three SVMs aided in increasing the specificity of predictions, which we consider more important than sensitivity at this stage of the search for unknown candidate miRNAs and their selection for experimental validation.

**Table 1:** Features used in mirCoS.

SVM model	Feature	F-score
SVM1	Ratio between number of positions with phastCons score ≥0.9 and number of positions with phastCons score ≥0.6 in the region	0.71
	Maximum phastCons score in the region	0.28
SVM2	Minimum free energy (MFE) for the predicted hairpin normalized by its length *	1.93 and 1.95
	Length of the hairpin*	0.63 and 0.46
	Fraction of the mouse hairpin sequence that overlaps with the human hairpin sequence in a net alignment of the genomes	0.45
	Predicted secondary structure conservation between the mouse hairpin and the most evolutionary distant genome its sequence aligns with	0.19
	GC content of the hairpin*	0.18 and 0.18
	Fraction hairpin bases that are in the stem*	0.08 and 0.09
SVM3	Fraction of miRNA bases that are paired in the hairpin	0.66
	MFE of the part of the hairpin that corresponds to the miRNA	0.63
	Number of bases in the predicted miRNA that are not conserved between human and mouse	0.59
	MFE of the part of the hairpin that is outside the predicted miRNA normalized by the length of that part	0.01

* Two values are given for features calculated separately for human and mouse.

We applied mirCoS to 976,746 regions from the mouse genome that are conserved in other vertebrates (conserved region set, CRS; see Methods). The CRS regions have a median size of 90 bp and cover 121,685,671 bp in total. Positive and negative examples are required to train SVMs and evaluate their performance. Our positive training examples for SVM1 consisted of all 310 regions in the CRS that overlapped known mouse miRNAs from miRBase release 9.1, and our positive training examples for SVM2 and SVM3 were derived from this set. Our negative training examples consisted of regions selected randomly from the rest of the CRS such that, for each training set and chromosome, the number of negative examples was the same as the number of positive examples. Because only a very low fraction of the mouse genome sequence is likely to encode miRNA, it is safe to assume that most negative training examples are not miRNAs. This can also be verified from the final result: only 0.3% of the regions in the CRS were classified as pre-miRNA. See Methods for further details about the construction of training sets.

For the selection of features to include in SVMs we used the F-score, which measures the discriminatory power of individual features. The F-score is related to the F-statistic used in analysis of variance, and has been shown to perform well in selecting features for SVMs [42]. Table 1 lists all selected features and their F-scores. A description of our rationale for choosing to evaluate these particular features follows.

**Figure 2:**
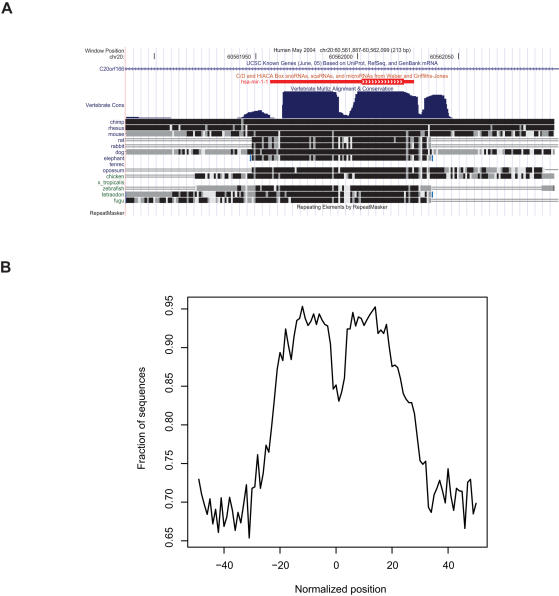
pre-miRNAs display a characteristic conservation profile. Typically, pre-miRNAs are highly conserved, but the conservation drops off rapidly at their borders and is often lower in the middle region, which corresponds to the loop. (A) Conservation profile of known pre-miRNA *hsa-mir-1-1* in the UCSC Genome browser (http://www.genome.ucsc.edu/). (B) Cumulative conservation profile of known mouse pre-miRNAs (from miRBase 8.2) conserved in human. Pre-miRNA regions were extended by 50 bp on each end and length-normalized to the range [-50,50]. The y-axis shows the fraction of analyzed sequences that are conserved at the position indicated on the x-axis.

To find appropriate features for describing the sequence conservation of pre-miRNAs, we inspected the vertebrate conservation track in the University of California Santa Cruz (UCSC) Genome Browser (http://www.genome.ucsc.edu/) (Figure 2A). Pre-miRNA genes are often highly conserved, but conservation drops off rapidly at their edges. Within pre-miRNAs, variations are more likely to occur in the central part of the conservation block, which corresponds to the loop part when the sequence is folded into a stem-loop structure. The generality of these observations is reflected in a cumulative human-mouse conservation profile based on all known mouse miRNAs that can be aligned to the human genome (Figure 2B). Similar, but less steep, miRNA conservation profiles have been distinguished in alignments of multiple primate species [8]. Consistent with this observation that most known pre-miRNA genes tend to stand out as islands of very high conservation in the genome, the best feature we could find for SVM1 was a measure of the ratio of high to intermediate conservation within a classified region (Table 1).

For a candidate region to be passed to SVM2, we required its predicted secondary structure to contain a pre-miRNA-like hairpin. All features in SVM2 relate to pre-miRNA hairpin structures. It has been shown that pre-miRNA hairpins, in contrast to other noncoding RNAs, have lower free energy of folding than randomized sequences with the same nucleotide content [43]. Accordingly, the best feature we found for SVM2 was normalized free energy of the predicted hairpin (Table 1). If a candidate region is a true pre-miRNA, its secondary structure should be conserved in all species where there is significant sequence conservation. As one of the features for SVM2, we therefore used predicted hairpin secondary structure conservation between each candidate mouse region and the most evolutionary distant genome that it could be aligned to, considering eleven vertebrate genomes at distances ranging from dog and cow to fish (see Methods).

For SVM3, the most discriminatory features–which were inspired by criteria successfully used for mature miRNA prediction in *C. elegans* [44]-measured the amount and conservation of base-pairing within the part of the predicted secondary structure corresponding to the miRNA. This is readily explained by the fact that mature miRNAs are always on the stems of hairpin structures, and the part of a stem that corresponds to a miRNA tends to have a high level of base pairing.

We tested the performance of SVM1 and SVM2 by jackknife cross-validation and obtained sensitivity estimates of 92% and 94%, respectively. For SVM3, since the number of positive examples was large, we used a different repeated holdout scheme to estimate its performance (see Methods) and obtained an average sensitivity of 85%.

### Prediction of 3400 miRNA genes conserved in sequence and structure

Application of mirCoS caused the number of candidate regions to decrease dramatically from 976,746 to 3476, while 68% of the known conserved miRNAs (from miRBase 9.1) that were used to train the model were retained. Table 2 shows the number of candidates and known miRNAs retained at each step of the prediction pipeline. Our final result set contained 3476 candidate pre-miRNAs from mouse and 3441 from human (Table 2; detail genome coordinates are in Dataset S1 and Dataset S2). Six percent of these candidates represent known pre-miRNAs from miRBase 9.1, while the remaining ones are putative novel pre-miRNAs.

**Table 2:** Number of candidates and known miRNAs maintained at each step of genome-wide screening.

	Mouse		Human	
	Candidates	Known miRNAs (miRBase 9.1)	Candidates	Known miRNAs (miRBase 9.1)
Before SVM1	976,746	310	820,001	298
After SVM1	389,018	283	384,937	272
After secondary structure prediction, and filtering out CDS and repeats	199,377	227	176,345	221
After SVM2	11,838	219	11,132	213
After SVM3	3,476	212	3,441	208

**Figure 3:**
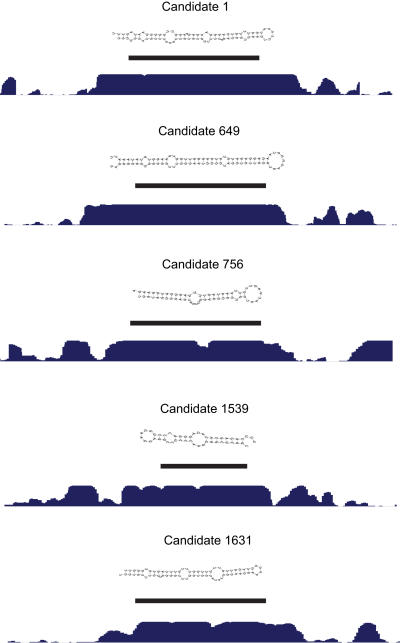
Predicted secondary structures and conservation profiles of five candidate pre-miRNA genes. The figure shows five examples from our predictions. Black bars indicate which regions of conservation profiles that correspond to predicted hairpins. Secondary structures of candidate pre-miRNAs were predicted by MFOLD v3.1 [63]. Conservation profiles were obtained from the UCSC Genome Browser (http://www.genome.ucsc.edu/). The candidates show canonical secondary structures and conservation profiles.

**Figure 4:**
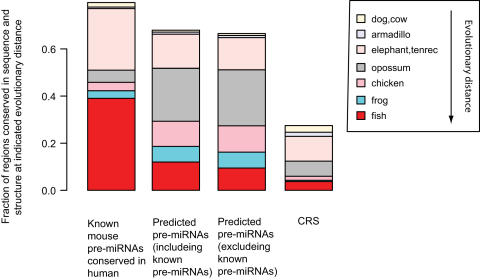
Candidate pre-miRNAs are conserved in sequence and predicted structure over large evolutionary distances. We used alignments between mouse and eleven other organisms to assess over what evolutionary distance each mouse region was conserved in both sequence and structure. Bars indicate what fraction of a particular set of regions that are conserved at a given distance. For each region, we only noted the most evolutionary distant species/clade at which we found it to be conserved. E.g. the leftmost gray bar spans 4%, indicating that 4% of known mouse miRNAs were found to be conserved in opossum, but not in chicken, frog or fish. The requirement for conservation was that regions should align over at least 37 nt and their predicted secondary structures have an RNAdistance score ≤48 (see Methods).

Five predictions of novel pre-miRNAs are illustrated in Figure 3. All these predictions have a very high level of conservation. To verify that the conservation constraints in the model worked well overall, we examined over what evolutionary distance our predictions were conserved in both sequence and structure, considering alignments to eleven vertebrate genomes as we did when computing input features for SVM2. Of our predictions for the mouse genome, 68% were aligned to the genome of an organism at an evolutionary distance ranging from dog and cow to (any of the) fish and showed predicted secondary structure conservation with that organism (Figure 4). Although this is lower than the result for known mouse pre-miRNAs conserved in human (80%), it is more than twofold the result of 27% for the initial set of candidate regions (the CRS) that were classified by SVM1. We obtained a very large enrichment for the farthest conservation examined, that with fish: 12% of predictions had fish conservation (either to zebrafish, *Tetraodon* or fugu), compared to only 3% of the CRS.

The sequence conservation features used in the model are based on phastCons scores, which primarily reflect patterns of base substitutions [45]. Lunter et al. [46] described a method to identify conserved regions based on rates of base insertions and deletions, and found that most known miRNA genes were within the identified conserved regions. Consistently, 80% of our candidate human miRNA genes overlap with conserved regions identified by Lunter et al. at 10% false discovery rate, compared to only 45% of the regions in the CRS and 3% of randomly selected regions.

Some of our novel candidates are homologs of known pre-miRNAs from other organisms. We BLASTed all our novel mouse candidates against all miRBase 9.1 pre-miRNAs using default blastn settings, and filtering the results to retain alignments (BLAST high-scoring segment pairs; HSPs) of length ≥50 bases and identity ≥75%. There were hits for five candidates: four hit known pre-miRNAs from human and the remaining candidate only hit two pre-miRNAs from chicken (gga-mir-147-1 and gga-mir-147-2). All these hits are likely true homologs, because sequence identity was high (mean: 91.3%, range: 87.1%–97.4%), as was the proportion of known pre-miRNA sequence aligned (mean: 87.0%, range: 58.3%–98.5%), strongly supporting that the five novel mouse candidates are true pre-miRNAs. Further analysis of the candidate that only had BLAST hits in chicken revealed that it was highly similar to mouse miRNA miR-147 cloned by Lagos-Quintana et al. [9]: 19/21 bases from the clone match. Importantly, the part of our candidate hairpin that aligned with miR-147 was within a 26 nt region predicted to contain a mature miRNA by our method. Mouse miR-147 had not been deposited in miRBase because it did not have a sufficiently good match to the mouse genome assembly. In the current mouse genome assembly (NCBI Build 36), the clone has five different matches with 19 identities each, but still no better match. However, Lagos-Quintana et al. found that their clone aligned with 20 identities to a region of the human genome predicted to form a hairpin structure. This region was subsequently deposited in miRBase as hsa-mir-147 based on the evidence from the mouse clone. The chicken miRNAs gga-mir-147-1 and gga-mir-147-2, which have not been experimentally validated, were annotated based on similarity (76% identity over 72 bases) to hsa-mir-147. Our novel human and mouse candidates are also similar to hsa-mir-147 (81% identity over 72 bases, although BLAST only found a 26-base HSP), but more similar to gga-mir-147-1/2 (87–89% identity over 70 bases). Experimental validation is required to show whether the mir-147 entries in miRBase, as well as our novel human and mouse candidates, represent true pre-miRNAs.

### Independent evidence supports the validity of miRNA predictions

To further assess the validity of our predictions, we examined several features that were not used in the prediction pipeline.

1. *Intronic vs. intergenic predictions -* The proportion of intronic miRNA genes is very similar between our predictions and known miRNAs: Of the 310 mouse miRNAs in miRBase 9.1 that are conserved (Table 2), we found 30% to be located within introns of protein-coding genes, compared to 35% for our candidates. For conserved human miRNAs, we found 35% of known and 37% of candidate miRNAs to be intronic (Table 3).

**Table 3:** Results from genome-wide screening for human and mouse miRNAs.

	Mouse	Human
Number of candidates	3476	3441
Number of known miRNA maintained	212	208
Number of intronic candidates	1219	1284
Number of intergenic candidates	1992	1879
Number of UTR candidates	265	278
Number of clusters	209	210

*2. Genomic clustering of predictions*-Many known miRNA genes occur in clusters along chromosomes. We identified spatial clusters among our predictions as described in Methods. Of our mouse candidates, 513 were clustered with one or more other candidates. The mouse candidates formed 209 clusters, 44 of which contained one or more known miRNA genes. Nineteen novel mouse candidates were clustered with a known miRNA gene, lending strong support to the validity of those predictions. The results for human were similar: 494 candidates formed a total of 210 clusters, 47 of which contained one or more known miRNA genes. Thirteen novel human candidates were clustered with a known miRNA.

**Figure 5:**
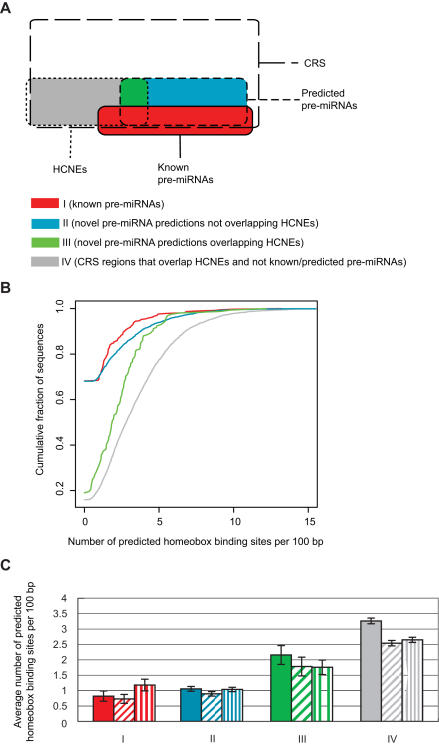
mirCoS can distinguish pre-miRNAs from highly conserved developmental enhancer regions. We compared differences in pattern composition among known pre-miRNAs, candidate pre-miRNAs and HCNEs. Each sequence was searched for putative transcription factor binding sites using the familial binding profile for homeobox transcription factors from the JASPAR database [72] at a score threshold of 80%. (A) Sequences were partitioned into four non-overlapping sets (I-IV) as indicated in the Venn diagram. (B) Cumulative distributions of number of predicted binding sites per 100 bp for sequence sets I-IV. The distributions for candidate pre-miRNAs (blue, green) are more similar to the distribution for known pre-miRNAs (red) than to the distribution for HCNEs not predicted to be pre-miRNAs (gray). (C) Solid bars show the average number of predicted sites per 100 bp over each of sequence sets I-IV. Shaded bars show results for corresponding control sets: controls for dinucleotide composition generated by, for each sequence, constructing a first-order Markov chain and using it to generate a new sequence (diagonal shading lines), and controls for single nucleotide composition generated by randomly shuffling the bases in each sequence (vertical shading lines). Error bars indicate 95% confidence intervals.

*3. Pattern composition*-Based on their genomic distribution, it was postulated that many highly conserved noncoding elements (HCNEs) may function as developmental enhancers [47]–[49]. Several have indeed been demonstrated to possess enhancer function (reviewed in [50]) and an enrichment of sequence patterns characteristic of binding sites for certain developmental transcription factors has been found in a large subset of HCNEs [51]. Since sequence patterns were not explicitly considered in mirCoS, we used pattern occurrence as an independent means to assess whether our model had discriminated between likely developmental enhancers and miRNA genes. For this analysis, we used a published HCNE set produced by scanning the human genome for regions with at least 95% sequence identity in mouse, as well as evidence of conservation in *Fugu* [48]. Figure 5 shows that HCNEs have a much greater incidence of putative binding sites for homeobox transcription factors than do known (p<10^−15^, Wilcoxon test) and candidate (p<10^−15^) miRNA genes. Figures for other transcription factors are in Figure S1. This difference in pattern composition can only be partially accounted for by differences in dinucleotide composition, suggesting that many of the binding sites predicted in HCNEs are functional (Figure 5C). Of our candidate pre-miRNAs, 221 (6%) overlapped with a HCNE. The occurrence of putative homeobox binding sites in the sequences for these 221 candidates was lower than for remaining HCNEs (p = 3×10^−7^, Wilcoxon test), indicating that mirCoS to some extent distinguishes between HCNEs that are miRNA genes and HCNEs that are developmental enhancers (Figure 5).

**Figure 6:**
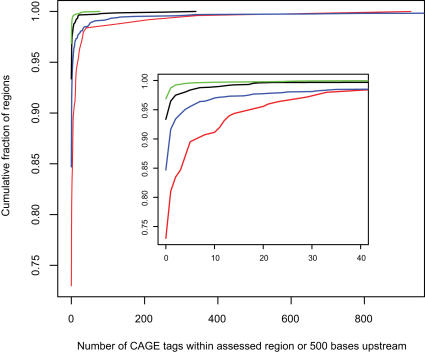
CAGE expression data supports miRNA predictions. Cumulative distribution of number of CAGE tags mapping to known intergenic pre-miRNA genes or within 500 bp upstream (red), and corresponding distributions for predicted intergenic pre-miRNAs (blue), randomly selected intergenic genomic regions of the same size (green) and intergenic regions from the CRS (black). Known and predicted pre-miRNAs tend to have more overlapping or upstream CAGE tags than either of the control sets. The inset shows a magnification for tags counts of 0–40.

*4. Evidence for transcription*-Some miRNA genes are known to be transcribed by RNA polymerase II as large primary miRNA transcripts which have cap structures and poly(A) tails [52], [53]. Additional support for the idea that intergenic miRNAs are transcribed as large primary miRNA transcripts comes from a recent study where the flanking genomic sequences (2.5 kb upstream and 4 kb downstream) of many intergenic mammalian miRNAs were found to align with expressed sequence tags (ESTs) [54]. In agreement with these results, we found EST and/or cDNA support for 59% of mouse miRNAs in miRBase 8.0 (the version used in [54]) and for 50% of mouse miRNAs miRBase 9.1 (Table 4). For our intergenic mouse miRNA predictions not represented in miRBase 9.1, we found EST/cDNA support for transcription of 345 (21%) of 1640. Corresponding rates for intergenic subsets of the CRS and randomly selected genomic regions are significantly lower (13% and 10%, respectively; p =  4×10^−9^ and p<10^−15^, respectively, compared to the rate for our predictions with chi-square tests) (Table 4) indicating that our set of miRNA predictions is enriched for transcribed sequences. The lower rate for our candidates compared to known miRNAs is expected, because most known miRNAs have been found by cloning methods, and miRNAs which have not yet been cloned are likely to be expressed at lower levels or in more restricted contexts. To more directly assess whether transcription start sites are present at or closely upstream of our miRNA predictions, we turned to cap analysis of gene expression (CAGE) data. CAGE is a technique to obtain sequence tags (CAGE tags) of about 20 nucleotides from 5′-ends of capped transcripts [55]. More than seven million CAGE tags have been sequenced from 145 mouse cDNA libraries and mapped to the mouse genome [56]. If primary miRNA transcripts have cap structures, their 5′-ends should be detectable by CAGE. We found 19% of known intergenic mouse pre-miRNAs to have more than one CAGE tag on the pre-miRNA or within 500 bp upstream, compared to 8% of our intergenic candidates (excluding known miRNAs, as for the cDNA/EST comparison above; genome coordinates of mouse and human candidates with CAGE data support are in Dataset S3 and Dataset S4). Corresponding rates for the CRS and randomly selected genomic regions are only 4% and 1%, respectively (Figure 6; p = 3×10^−8^ and p<10^−15^, respectively, compared to the rate for our predictions with chi-square tests). The difference in transcriptional support between miRBase 9.1 miRNAs and our novel predictions is similar between the comparison with cDNA/EST data and the comparison with CAGE data. However, the negative control sets (CRS and random genomic regions) have less support from CAGE than cDNA/EST data compared to known miRNAs and our predictions. The explanation may be that cDNAs and ESTs represent a variety of overlapping transcribed regions, while CAGE tags near genomic locations of pre-miRNA 5′-ends more specifically pinpoints transcriptional start sites for primary miRNA transcripts.

**Table 4:** cDNA and EST support for transcription of intergenic mouse miRNAs.

Support class a	mirBase 8.0	mirBase 9.1	Predictions b	CRS c	Random regions c
Entirely overlapped by cDNA/EST	33 (17.7%)	34 (13.7%)	79 (4.8%)	36 (2.4%)	31 (1.5%)
Partially overlapped by cDNA/EST	20 (10.8%)	24 (9.7%)	47 (2.9%)	42 (2.9%)	11 (0.5%)
In intron of cDNA/EST d	30 (16.1%)	35 (14.1%)	127 (7.7%)	62 (4.2%)	119 (5.6%)
In gap between 5′ and 3′ EST pair e	2 (1.1%)	2 (0.8%)	7 (0.4%)	1 (0.1%)	3 (0.1%)
Near unpaired EST f	25 (13.4%)	29 (11.7%)	85 (5.2%)	50 (3.4%)	50 (2.4%)
Sum	110 (59.1%)	124 (50.0%)	345 (21.0%)	191 (13.0%)	214 (10.1%)
All	186	248	1640	1470	2126

a Each known miRNA, prediction or other region was counted in one support class only, considering support classes in the order listed in the table.

b Intergenic miRNA predictions, excluding miRBase 9.1 miRNAs. Predictions with ambiguous orientation were randomly assigned to a strand for this test.

c CRS regions and random genomic regions were selected by sampling the same number of regions as there were miRNA predictions (3476), and then retaining only the intergenic regions.

d Regions counted in this table are intergenic with respect to UCSC known genes, but some are still in introns of poorly characterized cDNAs and ESTs. Only introns ≤50 kb and with canonical (GT..AG) splice junction sequences were considered.

e Gaps between ESTs were only considered if they spanned ≤50 kb.

f Based on the findings in [54], we considered as “near” 5′-ESTs within 2.5 kb upstream and 3′-ESTs within 4 kb downstream.

### Comparison to other methods

We compared our results to three other studies where SVMs were used to predict human or mouse miRNAs [37], [38], [41], as well as to two other recent studies where different techniques were used to predict miRNAs in the human or mouse genome [8], [57] (Table 5).

**Table 5:** Comparison between mirCoS and other methods.

	RNAmicro	RegEx	triplet-SVM	miR-abela	BayesMiRNAfind
Sequences searched	Human genome	Human genome	Region 56,000,001-57,000,000 of human Chr. 19 (assembly NCBI build 35)	10 kb upstream and downstream of mouse known miRNAs in miRBase 6.0	Forward strand of mouse genome
Number of candidates	5440 (3441)	976 (3441)	270 (4)	66 (46)	1697 (1731)
Number of known miRNA maintained	202 (208) from miRBase 9.1	191 (208) from miRBase 9.1	3 (1) from miRBase 5.0	22 (24) from miRBase 9.1	59 (111) from miRBase 6.0
Number of candidates shared with mirCoS	897	387	n.c.	15	n.c.

Numbers in parenthesis are results of mirCoS applied on the same sequences.

n.c., not calculated; we only computed overlap with mirCoS predictions for methods for which we could obtain the predictions.

Xue et al. [38] presented a method called triplet-SVM that recognizes pre-miRNAs based on their composition of small (3 nt) structure features. The method was trained on known human pre-miRNAs and achieved a high sensitivity (∼90%) when applied to known pre-miRNAs from human and several other organisms. Unlike mirCoS, triplet-SVM does not make use of cross-species information, and can therefore detect organism-specific miRNAs. However, triplet-SVM was not designed to be applied to whole genomes, even when combined with a conservation filter. As a specificity test, the authors applied it to human-mouse conserved segments from 1 Mb of human chromosome 19. The method classified 270 sequences from the 1 Mb region as putative pre-miRNAs, suggesting that it may predict several hundred thousand pre-miRNAs if applied to the entire human genome. Although triplet-SVM has a high sensitivity, its specificity in a whole-genome scan is therefore not comparable to ours.

Sewer et al. [37] also presented an SVM-based method (miR-abela) that does not make use of cross-species information. It appears that, with current knowledge of miRNA biology, such methods are unable to achieve the specificity required for whole-genome scans. Sewer et al. did not attempt a whole-genome scan, but applied miR-abela to detect clustered miRNA genes in human, mouse and rat genomic regions harboring known miRNAs. They detected 73 human and 51 mouse candidates that were not found by mirCoS, mainly because of our conservation constraints. Of these candidates, 12 and 10 represent known miRNAs (from miRBase 9.1), respectively. Conversely, we found 31 mouse and 26 human conserved miRNA candidates that miR-abela did not detect in the explored regions. Of these candidates, 11 and 10 represent known miRNAs in mouse or human, respectively. Thus, the two methods appear to complement each other well.

Recently, two methods have been described that detect conserved RNA secondary structures in alignments of multiple genomes [58], [59]. Hertel and Stadler [41] described an SVM-based method called RNAmicro, designed to detect pre-miRNAs in the output from such surveys. Like mirCoS, RNAmicro uses twelve different features for SVM classification. Although the exact choice of features differs, they relate to the same aspects of pre-miRNA: sequence, secondary structure and conservation. To compare the performance of mirCoS to RNAmicro, we obtained RNAmicro predictions for the human genome (J. Hertel, personal communication). Starting from conserved secondary structures detected by RNAz [59] and filtering the results at a score cutoff of 0.5, RNAmicro achieved sensitivity similar to our method: out of the 474 known human miRNAs in miRBase 9.1, RNAmicro detected 202 and we detected 208. However, at the same score cutoff of 0.5, RNAmicro predicted 58% more miRNA candidates in the human genome than mirCoS (5440 compared to 3441). From these counts, it appears that mirCoS has a higher specificity than RNAmicro, although RNAmicro specificity has been estimated to be high by comparison with a dataset of non-miRNA noncoding RNAs (J. Hertel, personal communication). The better performance of mirCoS is likely due to more stringent requirements in the model. RNAmicro asks for pre-miRNA secondary structures with stems of at least 10 bp, while mirCoS asks for secondary structures with a stems of at least 17 bp (the length of the shortest known mature miRNA). In mature miRNA prediction, RNAmicro mainly considers conservation of mature miRNAs, while mirCoS also considers base pairing (which is the most important criterion in MIRscan [44]) and minimum free energy of mature miRNAs. It is also interesting that the methods predict different candidates. Of the known miRNAs found by RNAmicro, we missed 34, and of those found by our method, RNAmicro missed 40. Comparing the entire candidate sets from the two methods, only 897 candidates (including 168 known miRNAs) are found in both sets. Predicted miRNAs are more likely to be true if clustered with other predicted or known miRNAs. In the human genome, 341 clusters were either created or expanded by adding our predictions to those from RNAmicro and known miRNAs.

Yousef et al. [57] used a different machine learning method, naïve Bayes classifier, to predict miRNAs conserved between human and mouse. They applied their method (BayesMiRNAfind) to the forward strand of the mouse genome sequence and presented results for different score cutoffs. At a cutoff that produced a similar number of miRNA gene predictions (1697) as mirCoS did on the forward strand of the mouse genome (1731), the Bayesian method detected 59 out of 135 known miRNA genes in miRBase 6.0. mirCoS found 111 of the same 135 miRNA genes, thus achieving a much higher sensitivity.

**Figure 7:**
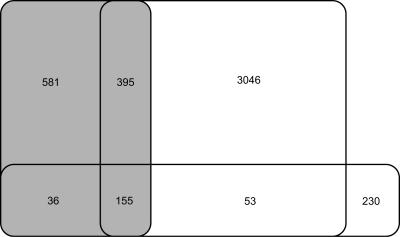
The overlap between our predictions and those from Berezikov et al. is small. Venn diagram showing the intersections between human miRNAs predicted by Berezikov et al.[8] (gray rectangle), our human predictions (large open rectangle) and known human miRNAs (horizontal rectangle).

Berezikov et al. [8] presented a method (here called RegEx) to detect miRNA genes by using a set of rules (implemented as regular expressions) to first scan human-rodent conservation profiles and subsequently predicted RNA secondary structures. The property of pre-miRNAs to have lower folding free energy than random sequences was also taken into account by filtering the results with the program Randfold [43]. We compared mirCoS with RegEx, because the features considered are similar and because, to our knowledge, RegEx represents one of the best miRNA prediction algorithms to date. On the current miRBase release (9.1), mirCoS has a somewhat higher sensitivity, recovering 208 known human miRNAs, among them 53 not found by RegEx, while RegEx recovered 191 known human miRNAs, among them 36 not found by us (Figure 7). Our higher sensitivity may be at the expense of specificity, because in total we predicted about three times more human miRNA genes than RegEx (3441 compared to 976). However, in addition to the 53 known conserved miRNAs missed by RegEx, our results contain many novel candidates that are highly likely to be true miRNAs and were missed by RegEx. Examples include two candidates clustered with known human miRNAs at chr14:100,411,114-100,411,204 and chr14:100,566,140-100,566,220 (coordinates refer to NCBI build 35). In total in the human genome, 196 clusters were either created or expanded by adding our predictions to those from RegEx and known miRNAs (from miRBase 9.1). Moreover, comparison with human CAGE data [56] indicates that mirCoS and RegEx have comparable specificity. We found 6.0% of our intergenic human candidates not represented in miRBase 9.1 to have more than one CAGE tag on the predicted pre-miRNA or within 500 bp upstream. The corresponding rate for intergenic RegEx predictions not represented in miRBase 9.1 was 6.1%, and rates for intergenic subsets of miRBase 9.1, the CRS and randomly selected regions were 9.4%, 2.0% and 0.6% respectively (these rates are lower than those given above for mouse, because less CAGE tags have been sequenced for human). To gain more insight into the differences between mirCoS and RegEx, we applied Randfold as an extra filter to our results, in the same way done in RegEx, and filtered out coding sequence and repeats from the results of RegEx, in the same way done in mirCoS. After this filtering, the trends in sensitivity and specificity remained, but were less pronounced: mirCoS found 181 known human miRNAs, compared to 176 for RegEx, and the total number of predictions was reduced to 1667 and 694, respectively, with 337 predictions shared between the two methods.

### Opportunities for improvements in miRNA prediction

Completion of the mammalian miRNA collection is likely to require an ensemble of approaches, including high-throughput sequencing, prediction of conserved miRNAs from multiple-species comparisons, and prediction of miRNAs that are not deeply conserved by analysis of single sequences. Each of these strategies has its drawbacks. Although high-throughput sequencing of small RNAs can identify numerous miRNAs with high specificity [60], miRNAs with highly restricted expression patterns may be difficult to detect even with massive sequencing capacity, especially in complex organisms. As demonstrated in this and earlier work [8], [41], cross-species conservation is highly informative for miRNA prediction. However, many recently discovered human miRNAs are not deeply conserved [7]. As discussed in the comparison with different methods above, current methods geared towards prediction of non-conserved miRNAs do not have sufficient specificity for searching large genomes. Very recently, two SVM-based methods for detecting miRNAs without use of cross-species comparisons were published [39], [40]. Despite innovative feature choices and significant improvements over previous methods, these new methods do not achieve sufficient specificity for application to the entire human genome, where they would predict on the order of ten or hundred thousand candidate miRNAs while recovering known miRNAs at a sensitvity of 85-90%.

All prediction methods are likely to benefit from the fact that new miRNAs are now continuously discovered and validated-with additional positive examples, prediction methods can be better trained. The integration of new genome sequences, miRNA target predictions and expression data into prediction pipelines will lead to further improvements. Moreover, the fact that there is little overlap among predictions from different top-performing miRNA prediction methods [41] suggests that much can be gained from combining strategies used in different methods. Finally, our knowledge of miRNA biology and biochemistry is advancing at a rapid rate: additional knowledge about miRNA biogenesis and target recognition is likely to aid the development of improved prediction strategies [39].

### Conclusions

We have developed a computational method–mirCoS-to predict the location of pre-miRNAs in genomic sequences. Unlike some other recent methods [37], [38], mirCoS is applicable to whole large genomes. Here, we have applied it to discover new miRNAs in the human and mouse genomes. The resulting predictions can be used to guide experiments that aim to clone and characterize new miRNAs. Importantly, we demonstrated that the performance of the method is very good. The predicted pre-miRNAs resemble known miRNAs in several aspects that were not considered by the prediction algorithm. The method outperforms another recently published method [57] in detecting human-mouse conserved miRNAs, and measures up to and complements other top-performing methods [8], [41] because there is little overlap among sets of miRNA candidates predicted by the different methods. Many of the predictions that we report, and that were not found by the methods we have compared with, are likely to represent real miRNAs, because many of them are located close to known miRNAs or miRNA predictions from other methods.

## Methods

### Sequence, expression and annotation data

Human genome assembly hg17 (NCBI Build 35), mouse genome assembly mm5 (NCBI Build 33) and annotations [cDNA-to-genome alignments, UCSC Known Gene coordinates, pseudogene and repeat locations, phastCons scores (calculated from multiple alignments of mm5, rn3, hg17, canFam1 and galGal2), pairwise net alignments between different genomes and multiple alignments of 16 vertebrate genomes with mouse (mm7, rn3, oryCun1, hg17, panTro1, rheMac1, canFam2, bosTau2, dasNov1, loxAfr1, echTel1, monDom2, galGal2, xenTro1, danRer3, tetNig1 and fr1)] were downloaded from the UCSC Genome Browser Database [61]. We obtained genomic coordinates of known miRNAs from miRBase [2], [3]. The CAGE data was produced in the FANTOM3 project [56] and can be downloaded at the FANTOM3 homepage (http://fantom.gsc.riken.go.jp). For the final version of the paper, the coordinates of predicted miRNAs have been lifted to NCBI Build 36 coordinates (hg18 and mm8).

### Construction of the conserved region set

By inspecting phastCons scores [45] for known mouse miRNAs from miRBase 7.1 in the UCSC Genome Browser, we observed that most mouse miRNAs either coincide with a distinct plateau of high conservation (Figure 2) or have very limited conservation. To construct the input to SVM1, we designed a set of rules for extracting conserved regions, so that the resulting set of conserved regions contained all known miRNAs that we by manual inspection found to have distinct conservation plateaus. We obtained two sets of conserved regions (0.14-regions and 0.5-regions) by scanning the mouse genome for maximal regions with phastCons scores above 0.14 and 0.5, respectively. Short (<37 bp) 0.14-regions were merged with any neighboring 0.14-regions less than 20 bp away. If exactly two 0.5-regions were within the same 0.14-region, we merged them. These merging steps were carried out because some miRNAs have a conservation drop in the part corresponding to the hairpin (Figure 2). If more than two 0.5-regions were within the same 0.14-region, we discarded them. Remaining (single or merged) 0.5-regions that spanned at least 37 bp constituted our set of candidate regions for SVM1. The threshold of 37 bp was chosen because it corresponded to the combination of the shortest known mouse mature miRNA (17 bp) and the shortest loop (3 bp). The conservation cutoffs were set to 0.14 and 0.5, because these were the maximal cutoffs at which all known mouse miRNAs with distinct conservation plateaus were included when requiring that the retained regions span at least 37 bp.

### Composite SVM model

We used LIBSVM tools (http://www.csie.ntu.edu.tw/∼cjlin/libsvm) to build the SVM models, with a radial basis function as kernel. Kernel parameters were selected by a grid search, using the scripts distributed with LIBSVM. The three SVMs were applied sequentially, as described in Results and Discussion. For each region that was classified as positive by SVM1, we extracted its best match in the human genome according to a net alignment between the two genomes [62]. We extracted hairpins that had a single loop and a stem of at least 17 bp from MFOLD v3.1 [63] secondary structure predictions for the human and mouse regions. We discarded all hairpins that in either genome overlapped with repeat annotation on the genome or CDS annotation in cDNA sequence (using all Genbank and RefSeq cDNAs mapped to the genome by UCSC as of May, 2004). Further, we only retained hairpins that were predicted in both organisms, i.e. on corresponding strands of aligned segments. To compute SVM2 and SVM3 features involving energy values, we obtained minimum free energy (MFE) values from MFOLD output, which contains the energy of each structure unit. We first computed an energy value for each nucleotide by dividing the energy of each structure unit (e.g. a base pair) equally among the nucleotides involved, and then computed feature values as the average energy per nucleotide considered for the feature (e.g. the first feature for SVM3 was computed as the average energy over the nucleotides in the predicted hairpin). To measure secondary structure conservation beyond rodents and hominids, we obtained a multiple alignment between mouse genome assembly mm7 (NCBI Build 35) and fifteen other vertebrate genomes from the UCSC Genome Browser Database [64]. We considered assemblies for eleven vertebrates in the following order: canFam2, bosTau2, dasNov1, loxAfr1, echTel1, monDom2, galGal2, xenTro1, danRer3, tetNig1 and fr1 (Figure 4). We used RNAfold to re-fold mouse hairpin sequences and their aligned sequences from other genomes, and computed conservation with RNAdistance [65]. To generate the features for SVM3, each hairpin classified as positive by SVM2 was scanned with a sliding window of length of 17nt (the size of the shortest known miRNA in mouse). To assess conservation of base pairs in the stems, we aligned human and mouse hairpins from corresponding strands and regions with LAGAN [66]. We trained one instance of SVM3 for human and one for mouse, and only retained candidates that were predicted as positive in both organisms. Following SVM3, we used the tRNAscan-SE Search Server (http://selab.janelia.org/tRNAscan-SE)[67] to eliminate putative transfer RNAs from the results. We also eliminated any predictions that overlapped with Vega [68] or Yale (http://www.pseudogene.org) pseudogene annotations in the human genome. Finally, because new protein-coding genes are still being discovered, we discarded all predictions that overlapped with CDS annotation from human and mouse UCSC Known Genes as of March, 2006 [69].

### SVM training and testing

Our set of positive examples for training SVM1 consisted of all regions in the candidate set that overlapped a known mouse miRNA (from miRBase 9.1), so that the overlap accounted for at least half of the length of the pre-miRNA. Our set of positive examples for training SVM2 consisted of all known mouse pre-miRNA hairpins retained after applying SVM1, and folding and filtering the sequences as described above. We obtained positive examples for training SVM3 by sliding a 17nt window across all known miRNAs in the hairpins retained after SVM2. For each SVM, negative examples were obtained by randomly sampling from the entire set of candidate regions that were retained after applying previous SVMs but did not represent known miRNAs. The sampling was done so that, for each training set and chromosome, the number of negative examples was the same as the number of positive examples.

**Figure 8:**
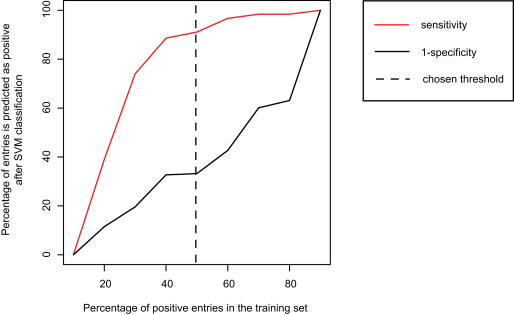
Effects of varying the proportion of positive entries in the training set for SVM1. Proportions of positive examples that were correctly classified (sensitivity, red line) and negative examples that were incorrectly classified (1-specificity, black line), as functions of the proportion of positive entries in the training set. Ideally, sensitivity should be maximized while 1-specificity should be minimized. From the figure, we can see that 1-specificity increases in three stages. At the beginning of the second increasing stage, sensitivity has already entered into a slowly increasing stage. We therefore chose this point (x = 50%, vertical line) as the proportion of positive entries to use in the training set.

To determine the optimal proportions of positive and negative examples for training SVM1, we randomly separated the positive examples into two sets of equal size. One of these was used to construct nine different training sets, by adding negative examples at a proportion ranging from 10% to 90%. We trained one SVM for each of these training sets, and used the remaining positive examples and the entire CRS as negative examples to evaluate SVM performance. Based on sensitivity and number regions classified as miRNA, we chose to use equal numbers of positive and negative examples in the final training set (Figure 8). Although we only performed this test on SVM1, we chose to use equal numbers of positive and negative examples for all three SVMs.

Because the number of known miRNAs is quite small, we did not separate them into distinct training and test sets. For SVM1 and SVM2, we instead validated performance by jackknife (a.k.a. leave-one-out) cross-validation. For SVM3, the number of positive examples was larger because of the sliding window approach used, and training SVM3 on all positive examples would have been too time-consuming. We therefore randomly divided them into 6 groups (for mouse) and 6 groups (for human), and added the same number of negative examples to each group. We trained one model with each of these groups, and tested each model on a different group. The cross-validation rate we report is the average over all groups. The training group with best sensitivity and smallest number of regions classified as positive was chosen as the training set for the final procedure.

### Feature selection

We used F-scores to measure the discriminatory power of each feature. The F-score is a simple measure of the discrimination of two sets of real numbers. Given training vectors *x_k_*, if the number of positive and negative instances are *n*_+_ and *n*_−_, respectively, then the F-score of the *i*th feature is defined as:
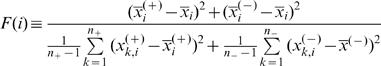
where *x̅* *_i_*, *x̅* *_i_* ^(+)^, *x̅* *_i_* ^(−)^are averages of the *i*th feature over the whole, positive, and negative data sets, respectively; *x_k,i_* ^(+)^ is the *i*th feature of the *k*th positive instance, and *x_k,i_* ^(−)^ is the *i*th feature of the *k*th negative instance. Larger F-scores indicate better discrimination [42]. We considered many features for each SVM. To determine which features to use in the final models, we ranked the features by F-score. Given *m* ranked features, we trained *m*-1 SVMs using features 1 and 2 for the first SVM, features 1, 2 and 3 for the second SVM etc. For feature selection, we judged SVM performance by training on the entire training sets, and studying the sensitivity and number of predicted miRNAs when the SVMs were applied to the entire set of candidates (see table S1). Table 1 lists all features used in the final SVMs.

### Analysis of pre-miRNA candidates

We classified miRNA gene candidates as intronic or intergenic according to their location relative to UCSC Known Genes [69]. We identified spatial miRNA gene clusters by clustering candidates predicted to be transcribed from the same strand. Intergenic candidates were clustered if they were separated by up to 6500 bp (the largest observed distance between two known clustered miRNAs), and intronic candidates if they were in the same intron. For transcription factor binding site searches, we used the TFBS Perl library [70]. To find support for miRNA expression, we downloaded all ESTs and and cDNAs mapped to mouse genome assembly NCBI 36 from the UCSC Genome Browser Database [61] in September 2006 and processed the mappings as described in [71] to remove low-quality sequences, reliably assign sequences to a genomic strand, and merge sequences obtained from the same cDNA clone. The selection of random regions for comparison with expression (cDNA, EST and CAGE) data was done so that these regions had similar chromosome and size distributions as the mouse miRNA predictions. Because comparisons of miRNA predictions and control sets to expression data were strand-specific, for the purpose of these comparisons, we randomly assigned strands to the randomly selected regions and the regions sampled from the CRS.

## Supporting Information

Figure S1 Pattern composition differences among known pre-miRNAs, candidate pre-miRNAs and HCNEs. Each sequence was searched for putative transcription factor binding sites using the familial binding profiles for HMG, ETS, Forkhead, MADS, REL, TRP cluster (MYB), bHLH(zip) and bZIP cEBP-like subclass transcription factors and binding profiles for pax6, nkx2.2, nkx6.1, gsh2 and oct from the JASPAR database [78] at a score threshold of 80%. All definitions and analysis are the same as what is described in the legend for Figure 4. In most cases, the distributions for candidate pre-miRNAs (blue, green) are more similar to the distribution for known pre-miRNAs (red) than to the distribution for HCNEs not predicted to be pre-miRNAs (gray).
(1.27 MB PDF)

Table S1 Process of feature selection for each SVM. Two tables are given for each SVM. The first table shows all tested features, ordered by F-score. The second table shows, for different feature sets, the number of input regions classified as positive by the model, and the number of known miRNAs (miRBase 7.1) classified as positive. Feature sets chosen for the final models are indicated in bold typeface and shaded in gray.
(0.05 MB DOC)

Dataset S1 Mouse candidate pre-miRNAs. File format: BED (see http://genome.ucsc.edu/FAQ/FAQformat) Description: Genome coordinates of all mouse candidate pre-miRNAs(Candidates 801 was deleted because of lifting to NCBI mouse genome assembly Build 36). The coordinates refer to the NCBI mouse genome assembly Build 36 (mm8).
(0.14 MB TXT)

Dataset S2 Human candidate pre-miRNAs. File format: BED. Description: Genome coordinates of all human candidate pre-miRNAs. The coordinates refer to the NCBI human genome assembly Build 36.1 (hg18).
(0.14 MB TXT)

Dataset S3 Mouse candidate pre-miRNAs with CAGE data support. File format: BED. Description: Genome coordinates of all mouse candidate pre-miRNAs with CAGE support. The coordinates refer to the NCBI mouse genome assembly Build 36 (mm8).
(0.01 MB TXT)

Dataset S4 Human candidate pre-miRNAs with CAGE data support. File format: BED. Description: Genome coordinates of all human candidate pre-miRNAs with CAGE support. The coordinates refer to the NCBI human genome assembly Build 36.1 (hg18).
(0.00 MB TXT)
