# Supplementary material for: Mammalian MicroRNA Prediction through a Support Vector Machine Model of Sequence and Structure
Source: PLoS One. 2007 Sep 26;2(9):e946. doi: 10.1371/journal.pone.0000946 (PMC1978525; doi:10.1371/journal.pone.0000946)
Supplement: Figure S1 — Pattern composition differences among known pre-miRNAs, candidate pre-miRNAs and HCNEs. Each sequence was searched for putative transcription factor binding sites using the familial binding profiles for HMG, ETS, Forkhead, MADS, REL, TRP cluster (MYB), bHLH(zip) and bZIP cEBP-like subclass transcription factors and binding profiles for pax6, nkx2.2, nkx6.1, gsh2 and oct from the JASPAR database [78] at a score threshold of 80%. All definitions and analysis are the same as what is described in the legend for Figure 4. In most cases, the distributions for candidate pre-miRNAs (blue, green) are more similar to the distribution for known pre-miRNAs (red) than to the distribution for HCNEs not predicted to be pre-miRNAs (gray). (1.27 MB PDF) [file pone.0000946.s001.pdf]

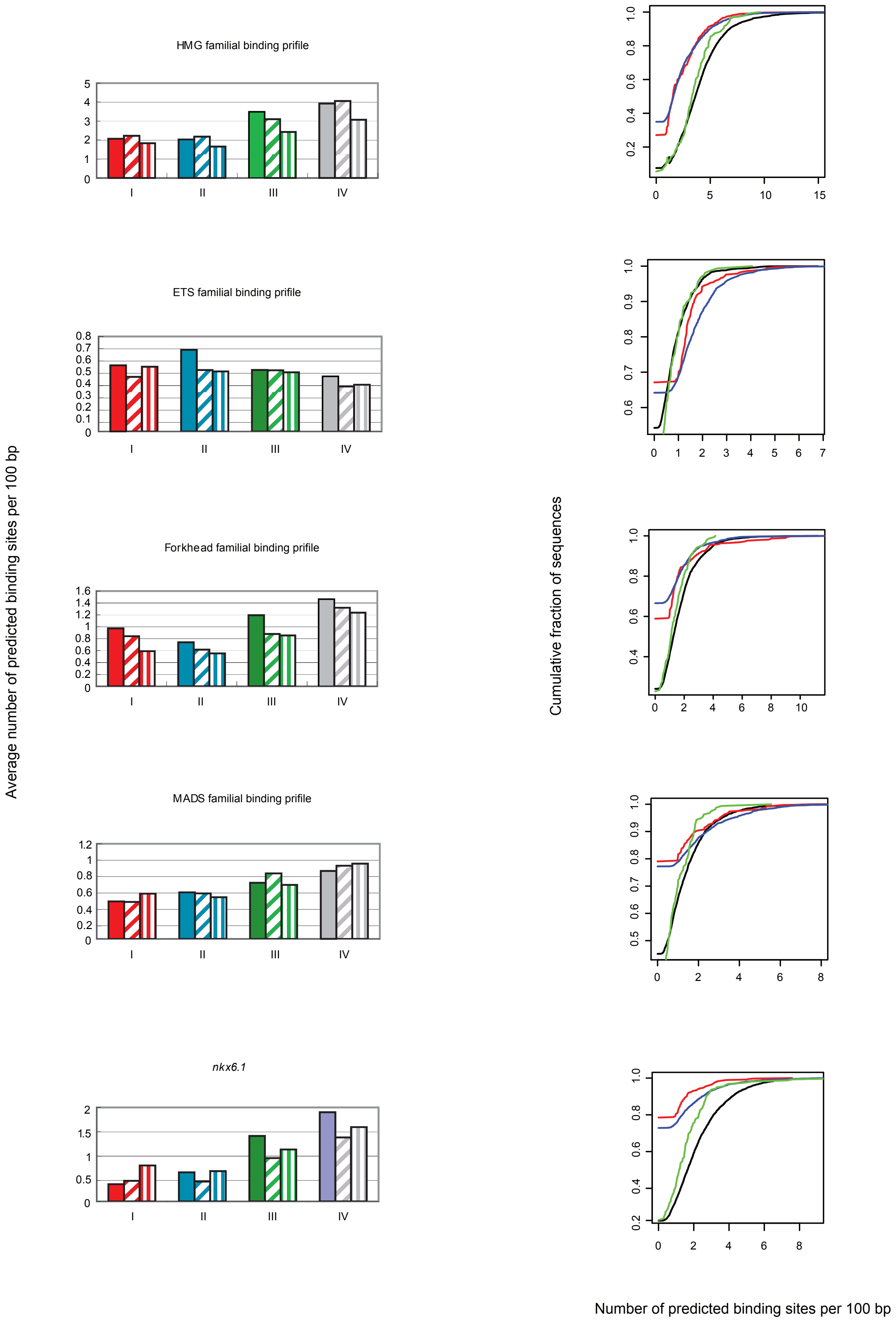
HMG familial binding prifile
ETS familial binding prifile
Forkhead familial binding prifile
MADS familial binding prifile
nkx6.1
I
II
III
IV
Average number of predicted binding sites per 100 bp
Cumulative fraction of sequences
Number of predicted binding sites per 100 bp

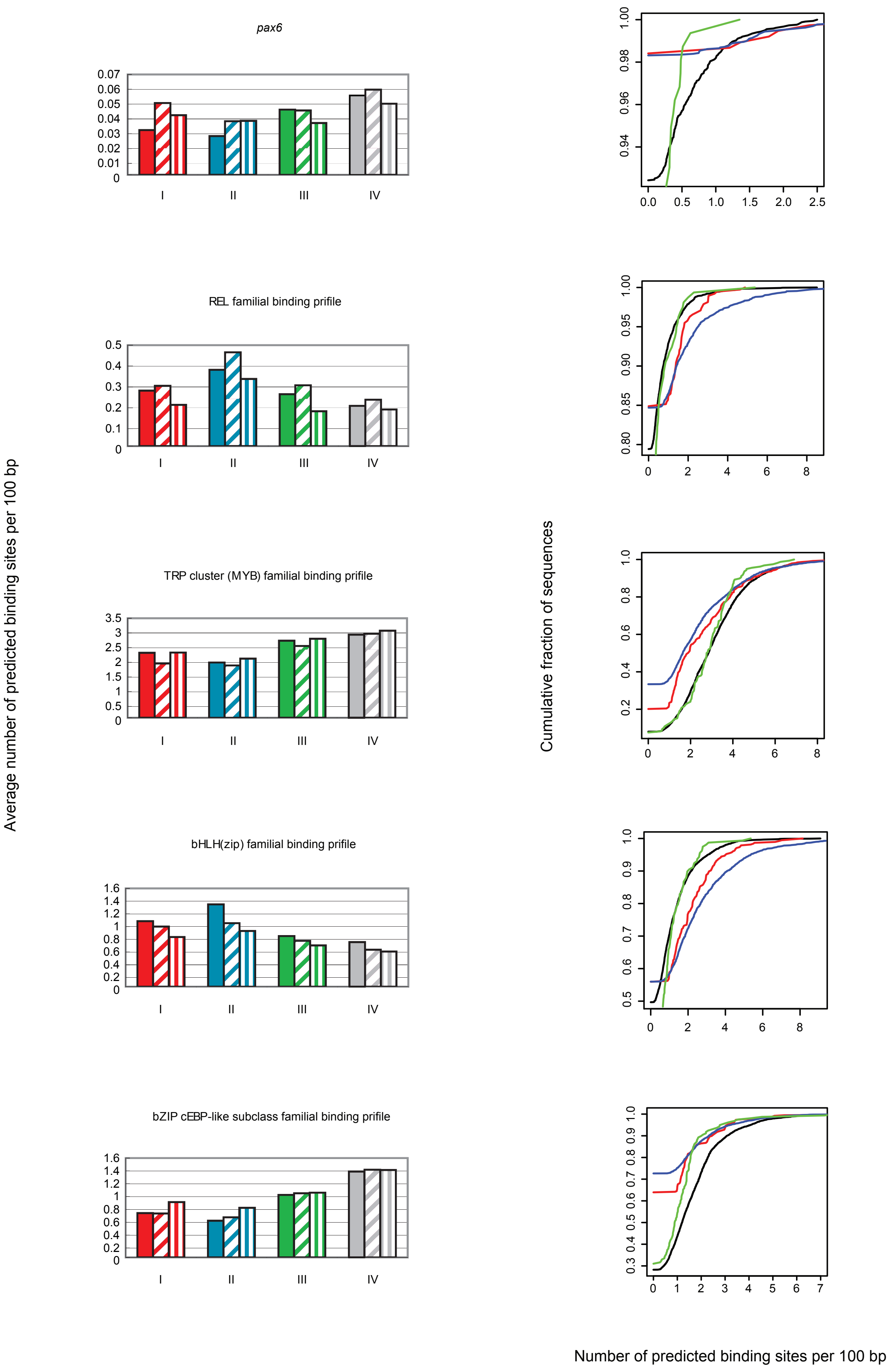

pax6
REL familial binding prifile
TRP cluster (MYB) familial binding prifile
bHLH(zip) familial binding prifile
bZIP cEBP-like subclass familial binding prifile
I
II
III
IV
Average number of predicted binding sites per 100 bp
Cumulative fraction of sequences
Number of predicted binding sites per 100 bp

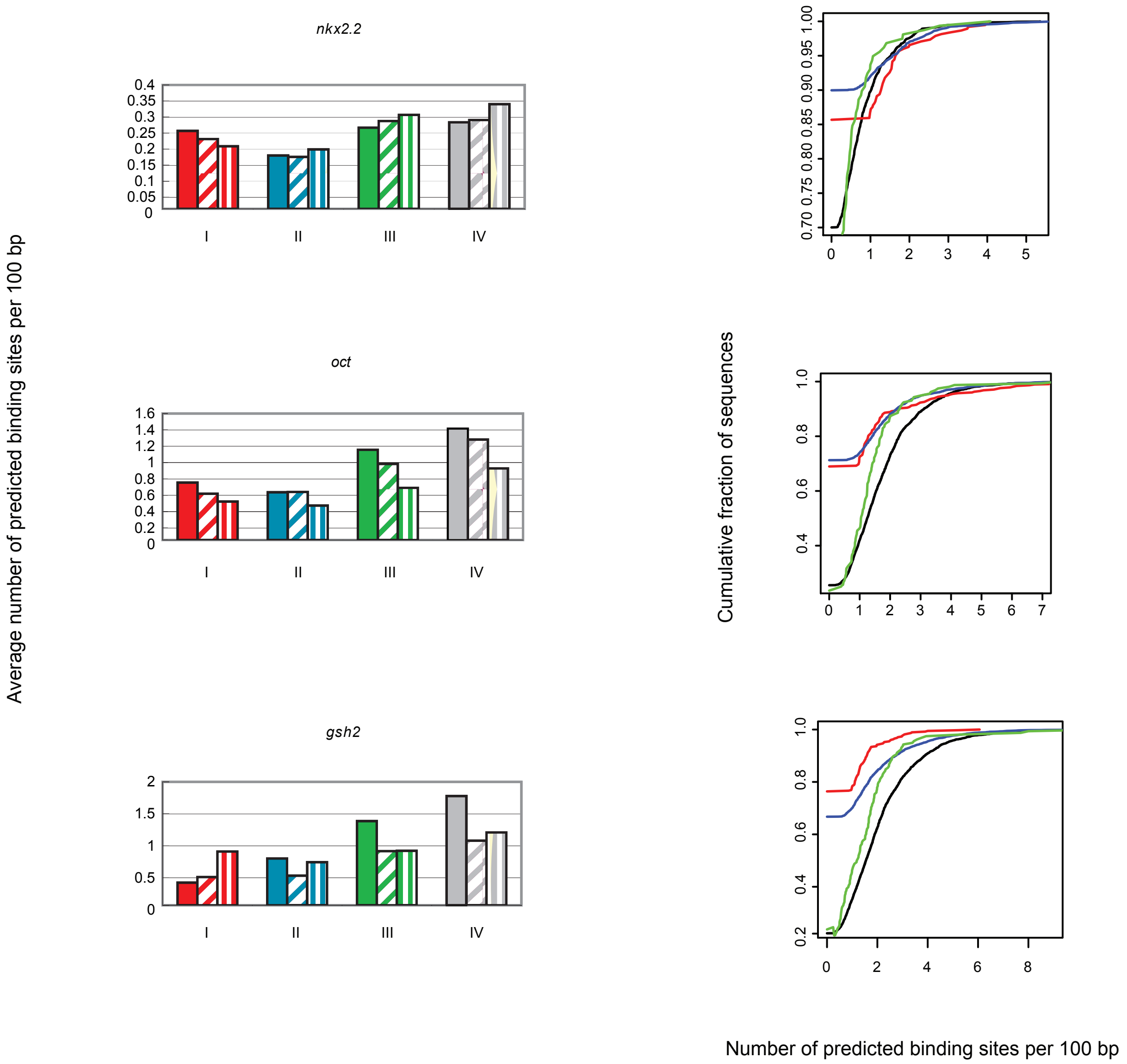
nkx2.2
oct
gsh2
I
II
III
IV
Average number of predicted binding sites per 100 bp
Cumulative fraction of sequences
Number of predicted binding sites per 100 bp
