# Supplementary material for: Mammalian MicroRNA Prediction through a Support Vector Machine Model of Sequence and Structure
Source: PLoS One. 2007 Sep 26;2(9):e946. doi: 10.1371/journal.pone.0000946 (PMC1978525; doi:10.1371/journal.pone.0000946)
Supplement: Table S1 — Process of feature selection for each SVM. Two tables are given for each SVM. The first table shows all tested features, ordered by F-score. The second table shows, for different feature sets, the number of input regions classified as positive by the model, and the number of known miRNAs (miRBase 7.1) classified as positive. Feature sets chosen for the final models are indicated in bold typeface and shaded in gray. (0.05 MB DOC) [file pone.0000946.s002.doc]

**Process of feature selection for each SVM**

**SVM1**

| **Features** | **F-score** |
| --- | --- |
| Ratio between number of positions with phastCons score ≥ 0.9 and number of positions with phastCons score ≥ 0.6 in the region (Feature 1) | 0.71 |
| Maximum phastCons score in the region(Feature 2) | 0.28 |
| Symmetry score. The region is divided into two parts by the center, and the symmetry score is calculated by summing up the absolute differences between phastCons scores at corresponding positions in left and right part (Feature 3) | 0.08 |
| Average edge difference. One 0.14-region contains one or two 0.5-regions. The average edge difference is computed as the average of two values: (1) the number of bases between the start of the 0.14-region and the start of the “leftmost” 0.5-region; and (2) the number of bases between the end of the “rightmost” 0.5-region and the end of the 0.14-region. (Feature 4) | 0.07 |
| The length of 0.5-region (Feature 5) | 0.05 |

| Features | Candidates | Known miRNAs  (miRBase 7.1) |
| --- | --- | --- |
| ***Feature1+Feature2*** | ***390280*** | ***283*** |
| Feature1+Feature2+Feature3 | 422746 | 285 |
| Feature1+Feature2+Feature3+Feature4 | 418316 | 284 |
| Feature1+Feature2+Feature3+Feature4+Feature5 | 420033 | 283 |

**SVM2**

| **Features** | **F-score** |
| --- | --- |
| Minimum free energy (MFE) for the predicted hairpin normalized by its length * (Feature 1) | 1.93 and 1.95 |
| Length of the hairpin* (Feature 2) | 0.63 and 0.46 |
| Fraction of the mouse hairpin sequence that overlaps with the human hairpin sequence in a net alignment of the genomes (Feature 3) | 0.45 |
| Predicted secondary structure conservation between the mouse hairpin and the most evolutionary distant genome its sequence aligns with (Feature 4) | 0.19 |
| GC content of the hairpin* (Feature 5) | 0.18 and 0.18 |
| Fraction hairpin bases that are in the stem* (Feature 6) | 0.08 and 0.09 |

| Features | Candidates | Known miRNAs  (miRBase 7.1) |
| --- | --- | --- |
| Feature1+Feature2 | 16032 | 224 |
| Feature1+Feature2+Feature3 | 17005 | 218 |
| Feature1+Feature2+Feature3+Feature4 | 17612 | 221 |
| Feature1+Feature2+Feature3+Feature4+Feature5 | 10864 | 218 |
| ***Feature1+Feature2+Feature3+Feature4+Feature5+Feature6*** | ***10606*** | ***218*** |

**SVM3**

| **Features** | **F-score** |
| --- | --- |
| Fraction of miRNA bases that are paired in the hairpin (Feature 1) | 0.66 |
| MFE of the part of the hairpin that corresponds to the miRNA (Feature 3) | 0.63 |
| Number of bases in the predicted miRNA that are not conserved between human and mouse (Feature 2) | 0.59 |
| MFE of the part of the hairpin that is outside the predicted miRNA normalized by the length of that part (Feature 4) | 0.01 |

| Features | Candidates | Known miRNAs  (miRBase 7.1) |
| --- | --- | --- |
| Feature1+Feature2 | 3920 | 212 |
| Feature1+Feature2+Feature3 | 3848 | 211 |
| ***Feature1+Feature2+Feature3+Feature4*** | ***3821*** | ***212*** |
